# A Novel Dual-Reporter System Reveals Distinct Characteristics of Exosome-Mediated Protein Secretion in Human Cells

**DOI:** 10.1186/s12575-023-00219-w

**Published:** 2023-09-20

**Authors:** Christopher Olson, Pengyang Zhang, Joy Ku, Renceh Flojo, Darin Boyes, Biao Lu

**Affiliations:** 1 Department of Bioengineering, Santa Clara University, 500 El Camino Real, Santa Clara, CA 95053 USA; 2 Department of Biology, Santa Clara University, 500 El Camino Real, Santa Clara, CA 95053 USA

**Keywords:** Exosome, Microvesicle, Extracellular vesicle, Dual-reporter system, Cell-to-cell communication, Bioluminescence luciferase, Fluorescence protein, Protein secretion

## Abstract

**Background:**

Exosomes, a special subtype of extracellular vesicles derived from human cells, serve as vital mediators of intercellular communication by transporting diverse bioactive cargos, including proteins and enzymes. However, the underlying mechanisms governing exosome secretion and regulation remain poorly understood. In this study, we employed a dual-reporter system consisting of bioluminescent Gaussia luciferase and fluorescent proteins to investigate the dynamics and regulation of exosome secretion in cultured human cells.

**Results:**

Our results demonstrated that the engineered dual-reporters effectively monitored both exosome-mediated and ER-Golgi-mediated secretory pathways in a specific and quantitative manner. Notably, we observed distinct characteristics of exosome-mediated protein secretion, including significantly lower capacity and different dynamics compared to the ER-Golgi pathway. This phenomenon was observed in human kidney 293T cells and liver HepG2 cells, emphasizing the conserved nature of exosome-mediated secretion across cell types. Furthermore, we investigated the impact of brefeldin A (BFA), an inhibitor of ER-to-Golgi membrane trafficking, on protein secretion. Interestingly, BFA inhibited protein secretion via the ER-Golgi pathway while stimulating exosome-mediated protein secretion under same experimental conditions.

**Conclusions:**

Collectively, our study highlights the utility of the dual-reporter system for real-time monitoring and quantitative analysis of protein secretion through conventional ER-Golgi and unconventional exosome pathways. Moreover, our findings unveil distinct features of exosome-mediated protein secretion, shedding light on its differential capacity, dynamics, and regulatory mechanisms compared to ER-Golgi-mediated proteins in human cells.

**Supplementary Information:**

The online version contains supplementary material available at 10.1186/s12575-023-00219-w.

## Background

Exosomes are an emerging intercellular communication system that can shuttle a range of bioactive cargos, such as proteins, enzymes, nucleic acids, and lipids between cells and tissues [1, 2]. In humans, exosomes are actively secreted by almost all cell types as nanoscale vesicles [3]. They exist ubiquitously in both the extracellular space and body fluids, and they utilize receptor-mediated endocytosis to deliver their cargo to recipient cells [3, 4]. Accumulated evidence has supported the hypothesis that exosomes play an important role in regulating numerous physiological and pathological processes, including tissue homeostasis [5], cell differentiation, organ development [6], viral infection and immune response [7, 8], cancer progression and metastasis [9], as well as cardiovascular [10] and neurological diseases [11]. The ability to actively secrete and deliver diverse bioactive molecules highlights their biological significance [3]. This unique characteristic has prompted the development of potential applications in translational medicine, For example, exosomes can be utilized as minimal liquid biopsy for disease diagnosis and prognosis [12], or as cell-free nano-therapeutics for targeted drug delivery and therapy [13]. Despite their importance, researchers currently lack effective tools to dynamically monitor the formation and release of exosomes in human cells. Advancements in this area would greatly contribute to both basic scientific knowledge and translational medicine.

Over the past decade, numerous research groups have investigated the biogenesis and secretion of exosomes in different cell types and organisms [14–16]. These studies have employed various traditional technologies such as electron microscopy [17], immunohistochemistry [18], and sequential ultracentrifugation [19] to identify intracellular organelles involved in exosome production. However, these traditional methods have limitations when it comes to real-time monitoring and quantification of exosome secretion. They are often time-consuming, low throughput, and do not provide dynamic information about the process. To overcome these limitations, several groups, including ours, have developed genetically encoded molecular reporters [20–23].

This study aims to accomplish two main objectives. First, it aims to design, construct, and validate new sets of dual-reporters that enable live monitoring and quantification of protein secretion through either conventional ER-Golgi-mediated or unconventional exosome-mediated pathways. Second, it intends to use these newly established tools to monitor and compare both pathways simultaneously, thereby identifying critical differences in their secretion mechanisms. To achieve these objectives, we have designed dual-reporters by fusing green/red fluorescence proteins (GFP/RFP) with Gaussia luciferase (gLuc). The design of the dual-reporters allows for the retention of the native signal peptide (SP) of gLuc, swapping the SP with a lipid anchor [24], or deleting the SP altogether. This approach results in the creation of two sets of dual-reporters that enable live monitoring of protein secretion through either the ER-Golgi-mediated pathway or the exosome-mediated pathway. The key feature of these dual-reporters is that they only differ in their signal peptide composition. By utilizing this new platform, we can conduct comparative studies on protein secretion through different secretory pathways.

In this study, we utilized lentivirus-based expression vectors to design and construct the dual-reporters. These vectors allowed for efficient expression of the reporters in mammalian cell culture systems. Using this newly developed platform, we conducted a comparative study on protein secretion in two different human cell lines: human kidney 293T and liver HepG2 cells. The results of this study demonstrated that exosome-mediated protein secretion has a lower capacity compared to the ER-Golgi-pathway. Additionally, we observed distinct dynamics and regulation of protein secretion between these two pathways. These findings provide valuable insights into the mechanisms of protein secretion and highlight important differences in the secretion processes mediated by exosomes and the ER-Golgi pathway.

## Materials and Methods

### Cells and Reagents

Human kidney 293T cells were purchased from Alstem Inc (Richmond, CA). Human liver HepG2 cells were purchased from ATCC (Manassas, VA). High glucose Dulbecco’s modified Eagle’s medium (DMEM) and Opti-MEM 1 × reduced serum media were purchased from Gibco (Billings, Montana). Ultraculture serum free media was purchased from Lonza (Hayward, CA). Fetal Bovine Serum (FBS) was purchased from HyClone Laboratories (Logan, UT). Polyethylenimine (PEI, Product No. 18978) was purchased from Millipore Sigma. Recombinant Gaussia princeps luciferase (CAT #321-100) was purchased from Nanolight Technologies (Pinetop, AZ). ExoQuick TC was purchased from System Biosciences (Palo Alto, CA). Brefeldin A (BFA) was purchased from eBioscience (San Diego, CA). Pierce™ Gaussia Luciferase Glow Assay Kit was purchased from Thermo Scientific (Waltham, MA).

### Human Cell Culture

HEK293T Cells and HepG2 Cells were maintained in 100 mm culture plates in DMEM media supplemented with 10% Fetal FBS and 100 U/ml Penicillin and 100 U/ml Streptomycin in a 37 C, 5% CO_2_ incubator. Cells were routinely passed at ratio of 1:4 ~ 6.

### Design Strategy and Construction of Fusion Reporters

**Fig. 1:**
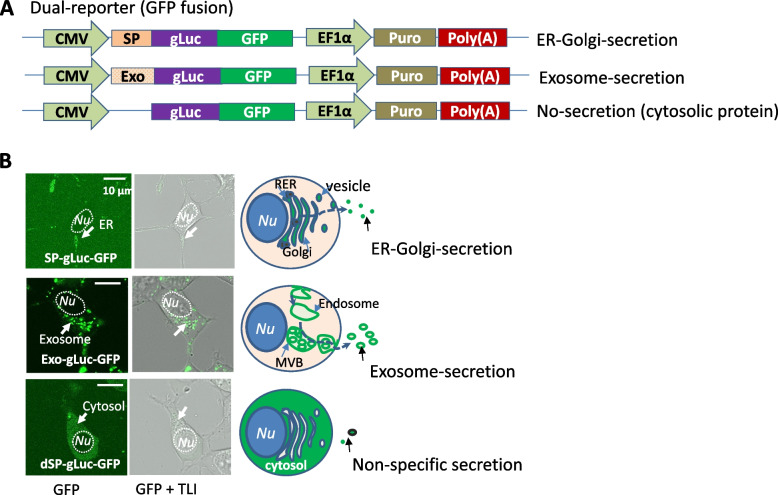
Configuration and construction of dual-reporters for visualization of secretory pathways in cultured human cells. Schematic illustration of key features of the dual-reporters, depicting dual-reporters of gLuc fused with GFP, under the control of CMV promoter (**A**). The functional validation of dual-reporters for monitoring of different secretory pathways of either exosome- or ER-Golgi-mediated secretion or non-specific secretion was conducted in 293T cells (**B**). Both fluorescence and transmitted light images were recorded 48 h after transfection with dual reporters. TLI: transmitted light image. Scale bar: 10 µm

Secretory pathway dual-reporters were a genetic fusion of green/red fluorescence (GFP/RFP) with Gaussia luciferase (gLuc) (Fig. 1A and Supplementary Fig. S1A). gLuc was tagged with either copepod GFP or ruby RFP at its C-terminus. The construction was carried out through PCR amplification of individual fragments and then joining amplified fragments together using a seamless cloning kit (SBI, Palo Alto, CA) [25]. These fusion genes (SP-gLuc-GFP/RFP) were then inserted into a lentiviral vector (CytoTracer, SBI) to yield the ER-Golgi constructs. Deletion of the signal peptide of gLuc (dSP-gLuc-GFP/RFP) or replacement of the endogenous signal peptide with an acylation peptide coding sequences (Exo-gLuc-GFP/RFP) was done using a service provided by Genscript. The reporter sequences and annotations are provided in the supplementary files (Supplementary sequences).

### Cell Transfection

Transfections were performed in 35 mm 4-Chamber Glass Bottom plates or 12-well plates. Typically, 293T or HepG2 cells were seeded and incubated overnight to reach 40–60% confluency. For transfection, the reporter DNA (1 μg/μl) and the transfection reagent polyethylenimine (PEI, 1 μg/μl) were mixed at a 1:5:100 ratio (volume: volume: volume in µl) in Opti-MEM. To facilitate DNA-PEI complex formation, plasmid and PEI were prepared separately, each with half of the Opti-MEM. The PEI solution was later combined with the plasmid solution and incubated at room temperature for 20 min before adding to the cell culture medium.

### Exosome Preparation and Purification

Exosomes were prepared and purified from the conditioned medium with a combination of centrifugation, ultrafiltration and chemical precipitation as described previously [26]. Briefly, 293T cultured on 150 mm culture plates were transfected with 20 µg plasmid for 24 h. Then, the transfection media was removed and replaced with Ultraculture to allow accumulation of exosomes in media for an additional 48 h. The exosomes were isolated from the conditioned medium with a combination of ultrafiltration, centrifugation and ExoQuick-TC precipitation.

### Nanoparticle Tracking Analysis

Nanoparticle tracking analysis (NTA) uses light scattering technology and Brownian motion for the determination of nanoparticle size in solution [23]. Typically, 1 ml of diluted exosomes was subjected to laser light scattering study. For individual exosome samples, three video recordings were obtained using a NanoSight LM10 instrument equipped with a 405 nm, 60 mV laser source. For imaging capture and detection, sCMOS camera was used with following equipment settings: the shutter length (30.012 ms), gain (500), and threshold (3-multi). Subsequently, NTA software was utilized to analyze and visualize exosome size and particle distributions. Exosome size and particle distributions were analyzed and graphed by the NTA software (NTA Version 2.3 Build 2.3.5.0033.7-Beta7).

### Live Cell Fluorescence Imaging and Confocal Microscopy

Cultured 293T and HepG2 cells were monitored and recorded using either a fluorescence LEICA DMI3000B microscope or a Leica TCS SP8 confocal system. Both fluorescence and transmitted light images were recorded for the same field. Image adjustments were applied to the entire image frame using Leica software. Colocalization study was carried out using ImageJ software program to assess the degree of colocalization via Pearson’s correlation coefficient analysis as reported [27].

### Brefeldin A Treatment

The treatment reagent, brefeldin A (BFA), was freshly prepared by diluting BFA stock solution (1000x) in our DMEM media to a 3 µg/mL working concentration. Transfected cells in 12-well plates were treated with BFA 24 h after transfection in the presence or absence of BFA (3 µg/mL) for 24 h. Gaussia luciferase activities were measured at indicated time-points.

### Gaussia Luciferase Assay

Gaussia luciferase activities inside transfected cells (cytosol) and outside of cells in the conditioned medium (secreted) were both quantified as reported previously [28]. Briefly, both conditioned media and transfected cells were collected and subject to Gaussia luciferase activity assays. Typically, 10 µL of either cell lysate or conditioned medium was then pipetted into microtiter wells. Luciferase activity was assessed immediately using a Tecan Infinite 200 Pro One plate reader. Recombinant Gaussia luciferase proteins were used to calibrate the plate reader equipment to ensure that measurements of all samples were within the linear range of detection.

### Data Analysis and Statistics

All experiments were repeated at least once unless otherwise indicated. Student’s two-tailed t-test was used to determine the statistical significance of our studies, with *p*-value < 0.05 being considered significant. Data and graphs were generated by Microsoft Excel software unless stated otherwise. We expressed the values from all measurements as mean ± standard deviation unless otherwise indicated.

## Results

### System Design and Experimental Approach for Monitoring Different Secretory Pathways in Living Human Cells

The ability to simultaneously visualize and quantify the temporal activity of protein secretion in living human cells is necessary to properly study the biological significance of different secretory mechanisms [29, 30]. To achieve this goal, we have designed and constructed two sets of dual-reporters via genetic fusion of gLuc to GFP or RFP. Like other molecular reporter systems designed for mammalian systems, a mammalian cytomegalovirus (CMV) promoter was used to drive reporter expression in human cells. To ensure appropriate subcellular targeting and secretion, the full-length gLuc with an endogenous signal peptide was kept at the N-terminus, while GFP/RFP was added at the C-terminus (upper panel, Fig. 1A and Supplementary Fig. S1A), which generated the SP-gLuc-GFP/RFP reporters. These two constructs were used to monitor the ER-Golgi-secretory pathway similarly to a previous report [31]. To direct the dual-reporter to exosomes, we replaced the 17 amino acid (aa) endogenous signal peptide of gLuc (MGVKVLFALICIAVAEA) with a 9-aa acylation peptide (MGCINSKRD) [24]. This modification resulted in two new constructs, Exo-gLuc-GFP/RFP, harboring the same dual-reporter proteins as of the ER-Golgi constructs (middle panel, Fig. 1A and Supplementary Fig. S1A). Our constructs also included two cytosolic reporters, created by deleting the N-terminus signal peptide of gLuc (cytosolic protein) to monitor non-secretion background or leakage [32], designated as dSP-gLuc-GFP/RFP (bottom panel, Fig. 1A and Supplementary Fig. S1A). This complete system will enable researchers to: (1) conduct comparative studies on conventional ER-Golgi and nonconventional exosome-mediated protein secretion with the same reporter format and vector system; (2) study exosome biogenesis, protein-loading, secretion, and intercellular communication visually and quantitatively; (3) use acid-resistant copepod GFP and ruby RFP, which are super bright fluorescent proteins with a fast maturation rate and stability in a wide range of temperatures and pH [33, 34], and suitable for tracing exosomes matured in an acidic environment.

### Dual-Reporters Enable Live Cell Monitoring of Both ER-Golgi and Exosome Pathways

We first assessed the gLuc-GFP/RFP reporters via fluorescence microscopy to test if they are suitable for secretory pathway monitoring in cultured 293T cells. Because our first set of constructs (SP-gLuc-GFP/RFP) contains the endogenous signal peptide (SP) at its N-terminus, we expected to observe the light-up of ER-Golgi and secretory vesicles with green fluorescence. As shown in Fig. 1B and Supplementary Fig. S1B (upper panel, arrows), the recorded images of transfected 293T cells exhibited punctuated GFP/RFP granules. These clustered fluorescent granules were situated at one side of the cytoplasm, consistent with an ER-Golgi distribution. For our second set of constructs (Exo-gLuc-GFP/RFP), the N-terminal acylation tag was able to target reporters to endocytic compartments [25], which were the biogenic sites of exosomes (Fig. 1B and Supplementary Fig. S1B; middle panel, arrows). These results suggest that Exo-gLuc-GFP may be targeted to pre-secreted exosome sites, such as multivesicular bodies (MVBs). Finally, our dSP-gLuc-GFP/RFP control constructs showed that the deletion of SP resulted in an even and diffused cytosolic accumulation of either dSP-gLuc-GFP or dSP-gLuc-RFP, which was consistent with their predicted cytosolic distribution (Fig. 1B and Supplementary Fig. S1B; bottom panel).

**Fig. 2:**
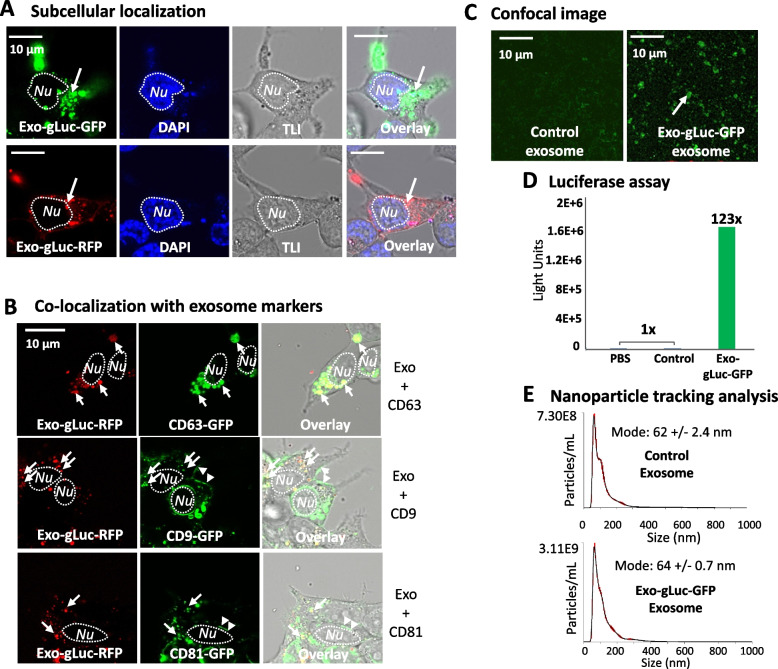
Live cell fluorescence imaging and trafficking of exosomes using dual-reporters. **A** Human 293T cells were transfected with Exo-gLuc-GFP and confocal images were recorded after 72 h of transfection. Images reveal that fluorescence signals (white arrows) were localized to cytosol, exhibiting punctate patterns. The nuclei of cells were stained blue by Hoechst reagent. **B** The colocalization of Exo-gLuc-RFP (red; white arrows) with exosome markers (green; white arrows: CD63-GFP, CD9-GFP, or CD81-GFP) in living human 293T cells was recorded after 72 h of transfection. The convergence of red and green fluorescence signals observed in the cytosol is demonstrated by yellow signals in the overly images (right column). **C** Confocal images of isolated exosomes from either non-modified control or Exo-gLuc-GFP transfected cells. **D** Gaussia luciferase activity was measured in PBS as well as in exosomes isolated from non-modified control and Exo-gLuc-GFP transfected cells. Data represents averages of two independent exosome preparations (*n* = 2). **E** Nanoparticle tracking analysis shows the vesicle size and distribution of exosomes isolated from either non-modified control and Exo-gLuc-GFP transfected cells. For each exosome sample, three video recordings were obtained using a NanoSight LM10 instrument equipped with a 405 nm, 60 mV laser source. NTA software was utilized to analyze and visualize exosome size and particle distributions. TLI: transmitted light image. Scale bar: 10 µm

To provide further evidence that our exosome reporters were targeted to the proper exosome-associated endocytic compartments, we subsequently confirmed the subcellular locations of the exosome reporters with high-definition confocal imaging. As shown in Fig. 2A, reporter constructs could light up the vesicle granules (green or red) accumulating in the cytosolic compartment, but not in the nucleus (stained in blue). The granular morphology and subcellular distribution of Exo-gLuc is consistent with endocytic compartments and exosome biogenesis sites as observed under the regular fluorescence microscope. It is worth mentioning that our exosome dual-reporters are primarily targeted to the endocytic membrane but not the plasma membrane [24]. To further these results, we conducted co-transfection experiments of our Exo-gLuc-RFP construct with three well-known exosome surface markers, including CD9-, CD63-, and CD81-GFP [35]. As expected, the Exo-gLuc-RFP reporters presented distinct and granular signal (red fluorescence) within the cytosol (Fig. 2B, left column), which matched the positive sites (green fluorescence) in all three exosome markers, as indicated by the convergence of yellow in overlay images (Fig. 2B, middle and right columns). We also conducted colocalization image analysis on the current micrographs to gauge colocalization with exosomal markers via Pearson’s correlation coefficient. Using the ImageJ program, we observed that exosome reporters exhibited stronger colocalization with CD63 (*r* = 0.733) compared to CD9 (*r* = 0.333) or CD81 (*r* = 0.297) [27]. These results support the notion that the N-terminal acylation tag can target dual-reporters to exosome biogenic sites during exosome formation, maturation, and eventual release into the extracellular space.

To confirm that the acylation tag can not only target gLuc to sites of exosome biogenesis but specifically load reporter proteins of gLuc-GFP/RFP into exosomes released into the extracellular space, we isolated exosomes from the conditioned medium of Exo-gLuc transfected cells and non-transfected control cells for confocal imaging, luciferase assay and nanoparticle tracking analysis (NTA). While only slight fluorescence background was found in the control samples, confocal images (Fig. 2C and Supplementary Fig. S1C arrows) showed robust fluorescence signals from both Exo-gLuc-GFP (green) and Exo-gLuc-RFP (red) respectively. Consistent with GFP/RFP positivity, isolated exosomes also demonstrated robust enhancements in luciferase activity in reporter-loaded exosomes as compared to control exosomes (Fig. 2D and Supplementary Fig. S1D). Together, these results indicate that our dual-reporters are successfully targeted to exosome compartments and subsequently released into the extracellular environment.

Next, we determined the size and particle distribution of our reporter-labeled exosomes as compared to non-modified controls. Our nanoparticle tracking analysis (NTA) data showed a single peak with a size range of 62–80 nm for exosome samples from either Exo-gLuc-GFP or Exo-gLuc-RFP, as well as 60–78 nm for non-engineered control exosomes, well within the expected size range for exosomes (50 ~ 150 nm). The mode size of reporter-loaded exosomes (64 ± 0.7 for Exo-gLuc-GFP and 79 ± 6.0 nm for Exo-gLuc-RFP) were slightly bigger than the non-loaded controls (62 ± 2.4 nm) (Fig. 2E and Supplementary Fig. S1E). These results are consistent with our recent report that genetic loading of protein cargos may increase the average size of exosomes [36].

### Exosome-Mediated Protein Secretion has a Low Capacity as Compared to the Conventional ER-Golgi Pathway

**Fig. 3:**
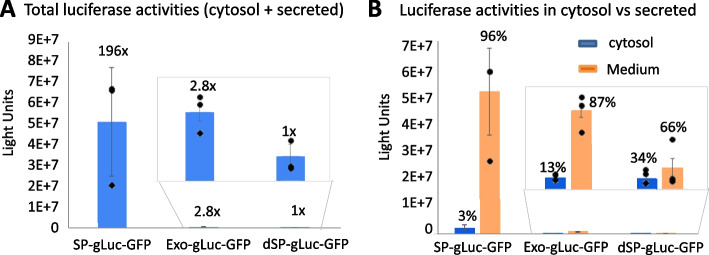
Secretory capacity of proteins via different pathways in cultured human cell. Human 293T cells were transfected with either ER-Golgi-pathway reporter (SP-gLuc-GFP), exosome-pathway reporter (Exo-gLuc-GFP), or non-secretory cytosolic protein as control (dSP-gLuc-GFP) for 48 h. Both cells and conditioned medium from the above three groups were collected and subjected to luciferase activity assay. The total activity from both cytosol (cell lysate) and secretion (conditioned medium) were graphed (**A** and **B**) (*n* = 3, mean ± SD). The luciferase enzyme remaining inside of producing cells versus luciferase enzyme secreted into the conditioned medium was graphed to show the relative amounts of luciferase production in both compartments as compared to the controls (*n* = 3, mean ± SD) (**C** and **D**)

To compare the capacity of protein secretion mediated by either the conventional ER-Golgi or the unconventional exosome pathways, we transfected 293T cells with reporter DNA (1 μg/well) (either SP-gLuc-GFP, Exo-gLuc-GFP, or dSP-gLuc-GFP), and subsequently measured luciferase activity 48 h after transfection. As shown in Fig. 3A, the levels of total luciferase activity (cytosol + secreted) were dramatically higher in SP-gLuc-GFP (196-fold) compared with that of the no-secretion control of dSP-gLuc-GFP (1 ×). As expected, the total luciferase activity of Exo-gLuc-GFP was also higher (2.8-fold) than that of the dSP-gLuc-GFP control (Fig. 3A). Strikingly, Exo-gLuc-GFP had a 70-fold lower level of luciferase activities than SP-gLuc-GFP, indicating that the exosome pathway has a much lower capacity for protein secretion. We graphed a comparison of the luciferase activity in the cytosol vs. conditioned media (Fig. 3B), revealing that the majority of luciferase protein was secreted into conditioned media, accounting for 96% and 87% of total luciferase activities for ER-Golgi (SP-gLuc-GFP) and exosome (Exo-gLuc-GFP) pathways, respectively. We opted to measure luciferase activity in the conditioned medium rather than isolated exosomes. This was due to the technical difficulties in isolating exosomes, especially in high throughput experiments and when dealing with minimal sample volumes. Although direct measurement within isolated exosomes could have strengthened our conclusions, we chose the conditioned medium approach for practical reasons.

**Fig. 4:**
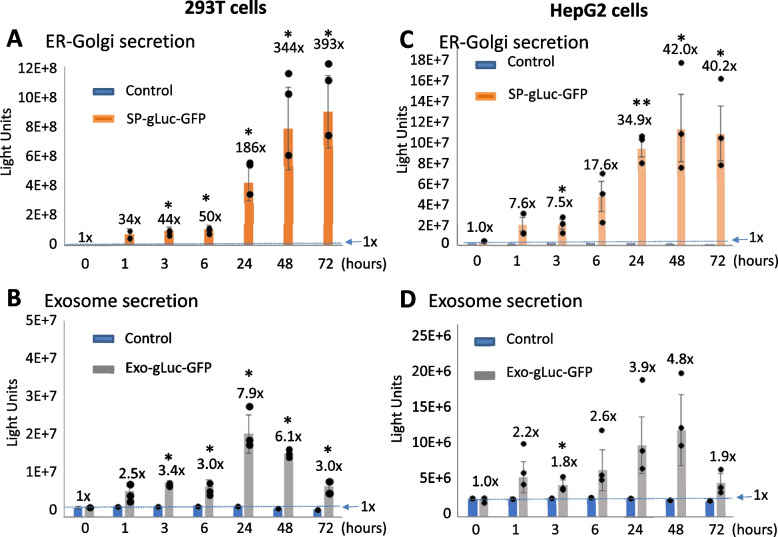
Comparative study on the secretory capacity and dynamics between ER-Golgi and exosome pathways. Human 293 T cells were transfected with either SP-gLuc-GFP or Exo-gLuc-GFP for 24 h. Gaussia enzyme secretion was monitored for up to 72 h at indicated time-points. The Gaussia luciferase activity was assayed and graphed (*n* = 3, mean ± SD) for ER-Golgi-reporter (**A**) and exosome-reporter (**B**). A similar time-course study was conducted in human HepG2 cells using the same protocol. The secretory dynamics were graphed (*n* = 3, mean ± SEM) for both ER-Golgi- and exosome-reporters respectively (**C** and **D**). Student’s two-tailed t-test was used to determine the statistical significance, with *p*-value < 0.05 being considered significant. * *P* < 0.05; ** *P* < 0.01

To further compare the secretory capability of both pathways, we conducted time-course studies on luciferase secretion in two different human cell lines, 293 T and HepG2. As shown in Fig. 4, the ER-Golgi pathway exhibited a steady and robust increase in the levels of luciferase activity in the conditioned medium of 293T cells at multiple time-points (1, 3, 6, 24, 48 and 72 h), which endured for a long period of time (Fig. 4A, SP-gLuc-GFP yellow bars vs. control blue bar). This increase started quickly 1 h after transfection (34-fold vs. non-transfection control), became significant at 3 h, climbed to 186-fold at 24 h, and reached its peak of 393-fold at 72 h. In contrast, the exosome pathway demonstrated relatively smaller but significant increases in the levels of luciferase activities in 293T cells (Fig. 4A, b, Exo-gLuc-GFP gray bars vs. control blue bar). Three hours after transfection, the luciferase activity significantly increased to 3.4-fold vs. control and reached its peak levels of 7.9-fold at 24 h, followed by a gradual decline at later time-points (48 and 72 h). These experiments suggest that the exosome pathway has a lower capacity and different dynamics/pattern of secretion as compared to the ER-Golgi pathway (Fig. 4A). To confirm our observations in human kidney 293T cells, we carried out a separate time-course study on human liver HepG2 cells using the same protocol. As shown in Fig. 4B, the liver cells revealed a similar trend of protein secretions mediated by these two pathways, albeit at a lower scale, with secreted luciferase activities of, on average, only ~ 1/5 of that in kidney cells. Based on these results, we concluded that unconventional exosome-mediated protein secretion differs from conventional ER-Golgi secretion in magnitude and dynamics.

### Exosome-Mediated Protein Secretion is Subject to Differential Regulation as Compared to ER-Golgi Pathway

**Fig. 5:**
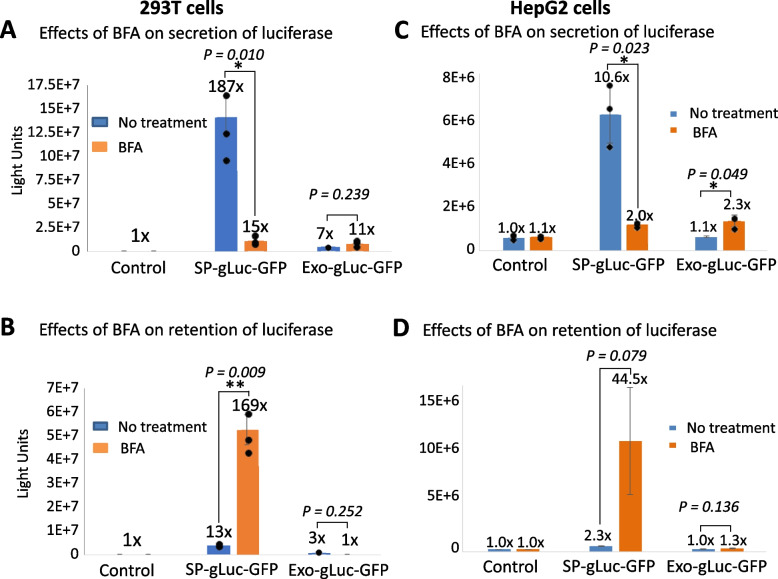
Differential regulation of protein secretion mediated by the ER-Golgi and exosome-pathways by BFA. Human 293T cells were transfected with either SP-gLuc-GFP or Exo-gLuc-GFP for 24 h. The transfected cells were treated with BFA reagent (3 µg/mL) for 24 h. Both conditioned culture medium and transfected cells were collected and subjected to luciferase assay. The secreted luciferase activity in the media from the transfected 293 T cells (**A**) and luciferase activity in 293T cells (**B**) were graphed (*n* = 3, mean ± SD). BFA experiments were also conducted in human HepG2 cells using the same protocol. The secreted luciferase activities in the medium (**C**), or luciferase activities in transfected cells (**D**) were graphed (*n* = 3, mean ± SD). Student’s two-tailed t-test was used to determine the statistical significance, with *p*-value < 0.05 being considered significant. * *P* < 0.05; ** *P* < 0.01

To gain more insight into potential differences in the control of protein secretion between these two pathways, we examined the effects of brefeldin-A (BFA) on luciferase secretion mediated by them. BFA is a well-known drug that is able to effectively inhibit ER-Golgi mediated protein secretion via blockage of ER to Golgi transition [37]. After transfection of human 293 T cells with the same amounts of reporter DNA for either ER-Golgi or exosome pathway for 24 h, we treated transfected cells with BFA (3 µg/mL). After 24 h of drug addition, we measured their luciferase activity of both conditioned media and transfected cells. As expected, while BFA treatment resulted in a drastic decrease in the levels of luciferase activity in media, equivalent to a 92% inhibition in ER-Golgi-pathway (middle bars, Fig. 5A). A corresponding increase in the levels of luciferase activity (a 13-fold vs. control) was observed in the cellular fraction (middle bars, Fig. 5B). In contrast, BFA treatment caused a small increase (~ 57% increase), rather than a decrease in the levels of luciferase activity in media in the exosome pathway (right bars, Fig. 5A). Correspondingly, BFA treatment also caused a moderate decrease (~ 67%) in the levels of cytosolic luciferase activity, suggesting a different mechanism of BFA regulation on the exosome pathway (right bars, Fig. 5B). To further confirm our observations in 293T cells, we conducted the same BFA experiments in human HepG2 cells. As shown in Fig. 5C and D, BFA treatment resulted in a similar inhibition of ER-Golgi-mediated luciferase secretion and a stimulation of exosome-mediated luciferase secretion, consistent with the results obtained from 293T cells. Together, our results demonstrate that the conventional ER-Golgi pathway and unconventional exosome-mediated pathway may be subject to differential controls and regulations.

## Discussion

Exosome-mediated protein secretion is becoming increasingly recognized as an important mechanism for mediating cell-to-cell communication in conjunction with the conventional ER-Golgi pathway [38]. However, little is known about exosome secretory capacity, dynamics, and regulatory control. The current methods used to study this unconventional exosome pathway, such as ultracentrifugation, immunohistochemistry, and radioactive tracing are complex, time-consuming, and have low throughput. Additionally, they lack the ability to analyze multiple pathways in a comparative manner [39]. In this study, we report the design, construction, and validation of a novel dual-reporter system to address these limitations. Dual-reporter systems allow for the visualization and quantification of protein secretion mediated by either the ER-Golgi pathway or the exosome pathway. Using this system, we can directly analyze several important physicochemical properties of protein secretion and gain insights into the capacity, dynamics and regulation of protein secretion controlled by these two pathways.

This study yielded important findings regarding the secretory capacity, dynamics, and regulatory controls of the ER-Golgi and exosome pathways. One notable finding is that ER-Golgi mediated protein secretion exhibits a much higher secretory capacity compared to the exosome pathway. The study also revealed differences in the patterns and dynamics of protein secretion between these two pathways. The time-course study demonstrated that the ER-Golgi pathway features rapid, steady, and robust secretion, reaching up to 393-fold increase over the control (Fig. 4A). Furthermore, protein secretion through the ER-Golgi pathway was sustained for at least 72 h. In contrast, exosome-secretion was less robust, reaching a 7.9-fold increase over the control at 24 h (Fig. 4A). After 24 h, the luciferase activity secreted by exosomes started to decline, indicating a different pattern of secretion compared to the ER-Golgi pathway (Fig. 4A-B). In addition to differences in capacity, pattern, and secretion dynamics, the study also uncovered differences in the regulatory controls of the two pathways. Treatment with BFA, a known inhibitor of ER-Golgi trafficking, drastically reduces luciferase secretion mediated by the ER-Golgi pathway (Fig. 5A and C). In contrast, BFA has an opposite effect on exosome-mediated luciferase secretion. This indicates a clear decoupling of these two pathways upon BFA treatment, suggesting different regulation mechanisms.

This study also produced some surprising findings, which may warrant future explorations. An intriguing observation is the marked disparity in total luciferase levels across these three cell model systems, despite identical backbone structure and CMV promoter. This discrepancy could stem from distinct post-translational modifications, differential degradation processes linked to specific subcellular localizations, or other factors. However, further investigation is required to unveil the precise mechanisms. Notably, the non-secretory control group demonstrates that 66% of total luciferase activity originates from post-transfection medium. We speculate that three possible explanations could account for this unexpected observation: (1) passive inclusion within exosomes or microvesicles during vesicle formation, (2) utilization of unknown secretory pathways, (3) membrane leakage due to experimental conditions such as transfection, or (4) a combination of these possibilities. Nonetheless, a thorough investigation is warranted to uncover the underlying mechanisms.

Exosomes are a special subset of extracellular vesicles (EVs) that also include microvesicles and apoptotic bodies [40]. Exosomes have an endocytic origin and have a diameter ranging from 30 to 150 nm [41]. In contrast, microvesicles are directly budded from the plasma membrane and have a larger and broader size range of 100 nm to 1 µm, while apoptotic bodies are released by dying cells and have a size range of 1 ~ 5 µm [42]. Due to the overlapping size and shared cellular machinery of these EV species, it has been challenging to develop reporter systems with high specificity for a single EV subtype. In a previous study by Shen B et al., they demonstrated that a simple 9-aa myristoylation tag can specifically target oligomeric cytoplasmic proteins to exosomes via a lipid anchoring mechanism. This finding suggested a potential new mechanism for highly specific modifications of exosomes [24]. Building upon this work, our study extends the application of the myristoylation tag to load Gaussia luciferase and dimeric copepod GFP or monomeric ruby RFP to exosomes. The results of our study support the myristoylation tag as a universal tag to specifically target various types of protein cargo (monomers, dimers and oligomers) to exosomes. Confocal imaging clearly shows that fluorescent signals are primarily located in endocytic compartments rather than the plasma membrane, consistent with exosome targeting rather than microvesicles (Fig. 1). Collectively, our work confirms the ability of this myristoylation tag to load different types of protein cargo into exosomes with high specificity. Recently, this lipid anchor was successfully used for functional loading of Cas9 enzymes into exosomes for gene editing applications [43]. We believe that this simple N-terminal tag, together with other exosome-targeting scaffolds, will serve as an important platform for the development of future exosome-based therapeutics [43–45]. It offers a promising scaffold for the specific loading of biologically active cargos into exosomes, facilitating the development of targeted and efficient exosome-based therapeutic strategies [46, 47].

### Supplementary Information

**Additional file 1.** Dual-reporter sequence related to this article.

**Additional file 2: Supplementary Figure S1.** Construction of dual-reporters and their characterizations.

## Data Availability

All data generated or analyzed in this study are included in this published articles and its supplementary sequences files. Data and experimental materials are available from the corresponding author upon reasonable request.

## Abbreviations

CMV: Cytomegalovirus promoter
gLuc: Gaussia luciferase
GFP: Green fluorescence protein
RFP: Red fluorescence protein
