# Supplementary material for: A Novel Dual-Reporter System Reveals Distinct Characteristics of Exosome-Mediated Protein Secretion in Human Cells
Source: Biol Proced Online. 2023 Sep 20;25:25. doi: 10.1186/s12575-023-00219-w (PMC10510171; doi:10.1186/s12575-023-00219-w)
Supplement: Supplementary file 1 — Additional file 1. Dual-reporter sequence related to this article. [file 12575_2023_219_MOESM1_ESM.pdf]

Protein and coding sequences of dual-reporters and sequence annotations

>SP-gLuc-GFP

MGVKVLFALICIAVAEAKPTENNEDFNIVAVASNFATTDLDADRGKLPGKKLPLEVLKEMEANARKAGCTRGCLICLSHIKCTPKMKK
FIPGRCHTYEGDKESAQGGIGEAIVDIPEIPGFKDLEPMEQFIAQVDLCVDCTTGCLKGLANVQCSDLLKKWLPQRCATFASKIQGQV
DKIKGAGGDHHHHHHNSNLNRIRGRMESDESGLPAMEIECRITGTLNGVEFELVGGGEGTPKQGRMTNKMKSTKGALTFSPYLLS
HVMGYGFYHFGTYPSGYENPFLHAINNGGYTNTRIEKYEDGGVLHVSFSYRYEAGRVIGDFKVVGTGFPEDSVIFTDKIIRSNATVEH
LHPMGDNVLVGSFARTFSLRDGGYYSFVVDSHMHFKSAIHPSILQNGGPMFAFRRVEELHSNTELGIVEYQHAFKTPIAFARSRAQS
SNSAVDGTAGPGSTGSR*

>Exo-gLuc-GFP

MGCINSKRKDKPTENNEDFNIVAVASNFATTDLDADRGKLPGKKLPLEVLKEMEANARKAGCTRGCLICLSHIKCTPKMKKFIPGRC
HTYEGDKESAQGGIGEAIVDIPEIPGFKDLEPMEQFIAQVDLCVDCTTGCLKGLANVQCSDLLKKWLPQRCATFASKIQGQVDKIKG
AGGDHHHHHHNSNLNRIRGRMESDESGLPAMEIECRITGTLNGVEFELVGGGEGTPKQGRMTNKMKSTKGALTFSPYLLSHVMG
YGFYHFGTYPSGYENPFLHAINNGGYTNTRIEKYEDGGVLHVSFSYRYEAGRVIGDFKVVGTGFPEDSVIFTDKIIRSNATVEHLHPMG
DNVLVGSFARTFSLRDGGYYSFVVDSHMHFKSAIHPSILQNGGPMFAFRRVEELHSNTELGIVEYQHAFKTPIAFARSRAQSSNSAV
DGTAGPGSTGSR*

>dSP-gLuc-GFP

MKPTENNEDFNIVAVASNFATTDLDADRGKLPGKKLPLEVLKEMEANARKAGCTRGCLICLSHIKCTPKMKKFIPGRCHTYEGDKES
AQGGIGEAIVDIPEIPGFKDLEPMEQFIAQVDLCVDCTTGCLKGLANVQCSDLLKKWLPQRCATFASKIQGQVDKIKGAGGDHHHH
HHNSNLNRIRGRMESDESGLPAMEIECRITGTLNGVEFELVGGGEGTPKQGRMTNKMKSTKGALTFSPYLLSHVMGYGFYHFGTY
PSGYENPFLHAINNGGYTNTRIEKYEDGGVLHVSFSYRYEAGRVIGDFKVVGTGFPEDSVIFTDKIIRSNATVEHLHPMGDNVLVGSF
ARTFSLRDGGYYSFVVDSHMHFKSAIHPSILQNGGPMFAFRRVEELHSNTELGIVEYQHAFKTPIAFARSRAQSSNSAVDGTAGPGS
TGSR*

>SP-gLuc-RFP

MGVKVLFALICIAVAEAKPTENNEDFNIVAVASNFATTDLDADRGKLPGKKLPLEVLKEMEANARKAGCTRGCLICLSHIKCTPKMKK
FIPGRCHTYEGDKESAQGGIGEAIVDIPEIPGFKDLEPMEQFIAQVDLCVDCTTGCLKGLANVQCSDLLKKWLPQRCATFASKIQGQV
DKIKGAGGDHHHHHHNSNLNRIRGRMGKLTMASSEDVIKEFMRFKVKMEGSVNGHEFEIEGEGEGRPYEGTQTAKLKVTKGGPLP
FSWDILSPQFQYGSKAYVKHPADIPDYLKLSFPEGFKWERFMNFEDGGVVTVTQDSTLQDGEFIYKVKLRGTNFPSDGPVMQKKT
MGWEASTERMYPEDGALKGEIKMRLKLKDGGHYDAEVKTTYKAKKQVQLPGAYMTDIKLDIISHNGDYTIVEQYERAEGRHSTGA
GSIIRSIIHHHHHH

>Exo-gLuc-RFP

MGCINSKRKDKPTENNEDFNIVAVASNFATTDLDADRGKLPGKKLPLEVLKEMEANARKAGCTRGCLICLSHIKCTPKMKKFIPGRC
HTYEGDKESAQGGIGEAIVDIPEIPGFKDLEPMEQFIAQVDLCVDCTTGCLKGLANVQCSDLLKKWLPQRCATFASKIQGQVDKIKG
AGGDHHHHHHNSNLNRIRGRMGKLTMASSEDVIKEFMRFKVKMEGSVNGHEFEIEGEGEGRPYEGTQTAKLKVTKGGPLPFSWD
ILSPQFQYGSKAYVKHPADIPDYLKLSFPEGFKWERFMNFEDGGVVTVTQDSTLQDGEFIYKVKLRGTNFPSDGPVMQKKTMGWE
ASTERMYPEDGALKGEIKMRLKLKDGGHYDAEVKTTYKAKKQVQLPGAYMTDIKLDIISHNGDYTIVEQYERAEGRHSTGAGSIIRSII
IHHHHHH*

>dSP-gLuc-RFP

MKPTENNEDFNIVAVASNFATTDLDADRGKLPGKKLPLEVLKEMEANARKAGCTRGCLICLSHIKCTPKMKKFIPGRCHTYEGDKES
AQGGIGEAIVDIPEIPGFKDLEPMEQFIAQVDLCVDCTTGCLKGLANVQCSDLLKKWLPQRCATFASKIQGQVDKIKGAGGDHHHH
HHNSNLNRIRGRMGKLTMASSEDVIKEFMRFKVKMEGSVNGHEFEIEGEGEGRPYEGTQTAKLKVTKGGPLPFSWDILSPQFQYGS

KAYVKHPADIPDYLKLSFPEGFKWERFMNFEDGGVVTVTQDSTLQDGEFIYKVKLRGTNFPSDGPVMQKKTMGWEASTERMYPE
DGALKGEIKMRLKLKDGGHYDAEVKTTYKAKKQVQLPGAYMTDIKLDIISHNGDYTIVEQYERAEGRHSTGAGSIIRSIIIHHHHHH*

Note we use the following color schemes for sequence annotation:

Yellow: signal peptide; Blue: acylation tag for exosome; Brown: Gaussia luciferase; Green: green fluorescence protein; Red: red fluorescence protein; Sequences without highlight: linker or epitope tag.
