# Supplementary material for: A Novel Dual-Reporter System Reveals Distinct Characteristics of Exosome-Mediated Protein Secretion in Human Cells
Source: Biol Proced Online. 2023 Sep 20;25:25. doi: 10.1186/s12575-023-00219-w (PMC10510171; doi:10.1186/s12575-023-00219-w)
Supplement: Supplementary file 2 — Additional file 2: Supplementary Figure S1. Construction of dual-reporters and their characterizations. [file 12575_2023_219_MOESM2_ESM.pptx]

## Slide 1
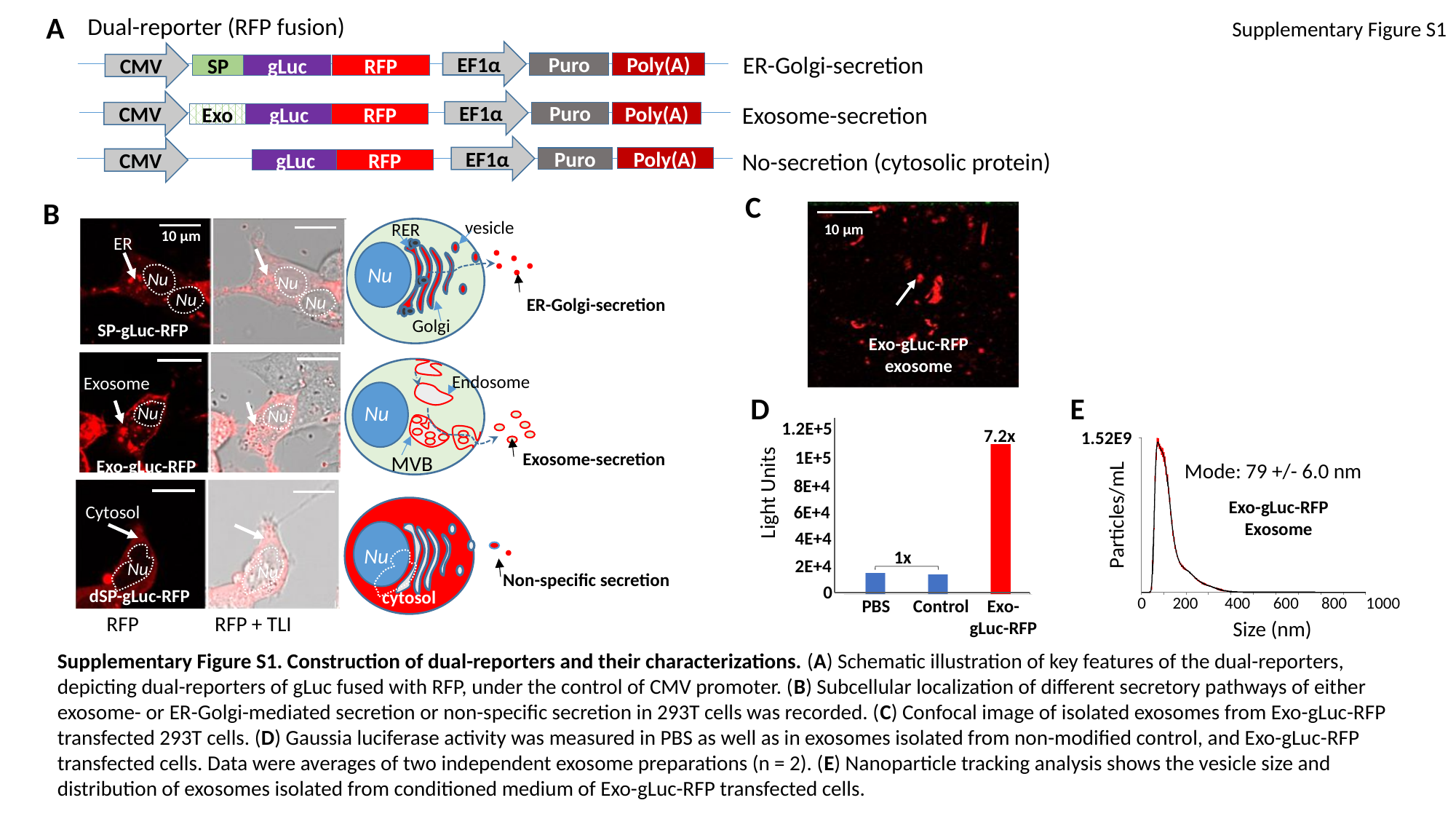

A
Dual-reporter (RFP fusion)
Supplementary Figure S1
EF1α
CMV
ER-Golgi-secretion
Poly(A)
Puro
SP
gLuc
RFP
EF1α
CMV
Exosome-secretion
Poly(A)
Puro
RFP
Exo
gLuc
EF1α
CMV
No-secretion (cytosolic protein)
Puro
Poly(A)
gLuc
RFP
C
B
vesicle
RER
ER-Golgi-secretion
Golgi
10 µm
ER
Nu
Nu
Nu
Nu
Nu
SP-gLuc-RFP
Endosome
Exosome-secretion
MVB
Exosome
Nu
Nu
Nu
Exo-gLuc-RFP
Cytosol
Non-specific secretion
cytosol
Nu
Nu
Nu
dSP-gLuc-RFP
RFP
RFP + TLI
Exo-gLuc-RFP
exosome
10 µm
D
E
1.2E+5
7.2x
1.52E9
Mode: 79 +/- 6.0 nm
Particles/mL
Exo-gLuc-RFP
Exosome
0 200 400 600 800 1000
Size (nm)
1E+5
Light Units
8E+4
6E+4
4E+4
1x
2E+4
0
PBS
Control
Exo-
gLuc-RFP
Supplementary Figure S1. Construction of dual-reporters and their characterizations. (A) Schematic illustration of key features of the dual-reporters, depicting dual-reporters of gLuc fused with RFP, under the control of CMV promoter. (B) Subcellular localization of different secretory pathways of either exosome- or ER-Golgi-mediated secretion or non-specific secretion in 293T cells was recorded. (C) Confocal image of isolated exosomes from Exo-gLuc-RFP transfected 293T cells. (D) Gaussia luciferase activity was measured in PBS as well as in exosomes isolated from non-modified control, and Exo-gLuc-RFP transfected cells. Data were averages of two independent exosome preparations (n = 2). (E) Nanoparticle tracking analysis shows the vesicle size and distribution of exosomes isolated from conditioned medium of Exo-gLuc-RFP transfected cells.
